# Incorporation of a Graduate Student Writer into a Productive Research Team

**DOI:** 10.5811/westjem.2016.9.31253

**Published:** 2016-11-08

**Authors:** Jonathan P. Fischer, Joseph B. House, Laura R. Hopson, Marcia A. Perry, Nikhil Theyyuni, Margaret S. Wolff, Cemal B. Sozener, Sally A. Santen

**Affiliations:** * Alumnus of University of Michigan, School of Public Health, Ann Arbor, Michigan; † University of Michigan, Department of Emergency Medicine, Ann Arbor, Michigan; ‡ University of Chicago Pritzker School of Medicine, Chicago, Illinois; § University of Michigan, Department of Learning Health Sciences, Ann Arbor, Michigan

## BACKGROUND

An academic physician is faced with the unique challenge of balancing clinical practice with demanding education and research obligations. These competing tasks often result in a lack of time dedicated to research, which can result in incomplete and unpublished projects. One study found that abstracts presented at emergency medicine conferences were subsequently published only 23%–47% of the time.^1^ Another study found similar results of only 33%.^2^ A lack of time is cited as the primary reason physicians do not prepare more papers for publication.^3^ Although some physicians overcome this hurdle through professional writing companies, this practice is discouraged in the academic field.^4^ Given the importance of publications to faculty for promotion and the community of educators for advancing their practice, we sought to create a more productive research model that reduces the time burden of manuscript preparation for busy teams of physicians.^5^, ^6^

## OBJECTIVES

The objective of this innovation is to describe a novel approach to assist with scholarly productivity by intentionally incorporating a graduate student research assistant (GSRA) into a research team to help with manuscript preparation.

## INNOVATION DESIGN

The Medical Education Research Group (MERG) at the University of Michigan, the structure of which has been described elsewhere, created the position of a GSRA as part of its research team in 2013.^5^ Student research assistants commonly engage in data collection in the emergency department, but they much less frequently assist in publication. The idea behind the GSRA was that time constraints made it difficult for many members of MERG to complete the process of moving abstracts to manuscript preparation. Graduate students, on the other hand, are frequently required during their coursework to gather resources about a topic they have not previously been exposed to, synthesize the information, and produce a term paper. Thus, MERG leadership postulated that successful GSRAs could apply the same skills they use for their courses to assist physicians in bringing projects to completion.

A master’s student from the University of Michigan’s School of Public Health was hired as a GSRA, and was paid $15 per hour for approximately 10 hours per week. Half of this funding came from the federal Work-Study Program and half of it through departmental funding. The GSRA was supervised by the leader of MERG, who spent about two hours per month on this task. Steps of integrating the GSRA were as follows: First, the GSRA provided input into data analysis, interpretation, and determining the scope of the project. Second, the GSRA conducted a literature review and began working on writing the introduction with the first author. Meanwhile, another member of the team produced a draft of the methods and results sections. Third, the research team as a whole discussed ideas for how to frame the paper, which relevant background topics to include, and what conclusions should be drawn. Fourth, the GSRA organized these ideas, fit them into the framework of the existing literature on the topic, and completed a draft of the introduction, discussion, and conclusion sections for a paper. Finally, these sections were disseminated to the entire team, who actively revised them. All of the GSRA’s projects involved preexisting data or data that were being collected. Because of this, the GSRA had a rapid turnaround of projects and completed about one per month. Since the GSRA made significant contributions during each of these steps, from clarification of the research question to background data interpretation to initial writing and final revision, the GSRA met authorship requirements for each project.^7^

## IMPACT & EFFECTIVENESS

The addition of a GSRA was associated with more rapid project completion and paper submission. During the first academic year, seven papers were completed with the GSRA. Five of them have since been published, one was published as an abstract and is being edited for resubmission, and one is unpublished and is being edited for resubmission.^5^, ^8^^–12^ The GSRA also provided minor assistance on an additional project that has not been published. Therefore, at a cost of <2,000 dollars, the GSRA helped publish five papers. Precise measures of increased productivity remain unknown; however, MERG members reported reduced time to paper submission.

A key to the success of MERG has been maintaining a high degree of structure with well-defined roles, regular meetings, and committed leadership. We found that a GSRA was a cost-effective and productive addition to our team. A GSRA is likely not appropriate for some research groups though. This model may not increase productivity when limiting factors beyond time constraints exist, such as a lesser robustness of data, lack of research mentorship, or motivation on the part of faculty. In addition, many groups may find it difficult to recruit graduate students with interests in both writing and medical education. Finally, our results are not generalizable beyond structured research teams, as it remains unknown what effect a GSRA could have when used with individual physicians. In teams such as MERG, however, our experiences show that a GSRA could provide valuable writing assistance and lead to more efficient research output.
